# Modes of Accessing Bicarbonate for the Regulation of Membrane Guanylate Cyclase (ROS-GC) in Retinal Rods and Cones

**DOI:** 10.1523/ENEURO.0393-18.2019

**Published:** 2019-02-15

**Authors:** Clint L. Makino, Teresa Duda, Alexandre Pertzev, Tomoki Isayama, Polina Geva, Michael A. Sandberg, Rameshwar K. Sharma

**Affiliations:** 1 Department of Physiology and Biophysics, Boston University School of Medicine, Boston, MA 02118; 2 Research Divisions of Biochemistry and Molecular Biology, Unit of Regulatory and Molecular Biology, Salus University, Elkins Park, PA 19027; 3 Department of Ophthalmology, Harvard Medical School, Boston, MA 02114

**Keywords:** bicarbonate, cone, receptor guanylate cyclase, retina, rod, visual transduction

## Abstract

The membrane guanylate cyclase, ROS-GC, that synthesizes cyclic GMP for use as a second messenger for visual transduction in retinal rods and cones, is stimulated by bicarbonate. Bicarbonate acts directly on ROS-GC1, because it enhanced the enzymatic activity of a purified, recombinant fragment of bovine ROS-GC1 consisting solely of the core catalytic domain. Moreover, recombinant ROS-GC1 proved to be a true sensor of bicarbonate, rather than a sensor for CO_2_. Access to bicarbonate differed in rods and cones of larval salamander, *Ambystoma tigrinum*, of unknown sex. In rods, bicarbonate entered at the synapse and diffused to the outer segment, where it was removed by Cl^-^-dependent exchange. In contrast, cones generated bicarbonate internally from endogenous CO_2_ or from exogenous CO_2_ that was present in extracellular solutions of bicarbonate. Bicarbonate production from both sources of CO_2_ was blocked by the carbonic anhydrase inhibitor, acetazolamide. Carbonic anhydrase II expression was verified immunohistochemically in cones but not in rods. In addition, cones acquired bicarbonate at their outer segments as well as at their inner segments. The multiple pathways for access in cones may support greater uptake of bicarbonate than in rods and buffer changes in its intracellular concentration.

## Significance Statement

Bicarbonate accentuates the role of the membrane guanylate cyclase, ROS-GC, in phototransduction. In current research, bicarbonate rather than gaseous CO_2_ is proven to be the ligand for ROS-GCs. Bicarbonate may be preferred because its movements are subject to tighter control. In rods, membrane impermeant bicarbonate gains entry at the synaptic zone and is cleared by Cl^-^-dependent exchange at the outer segment. A novel insight is that while cones are similar, they also obtain bicarbonate at their outer segments and express an enzyme for making their own bicarbonate from endogenous together with exogenous CO_2_. Multiple pathways for access in cones indicate a need for greater uptake of bicarbonate and better buffering of its intracellular concentration than in rods.

## Introduction

Bicarbonate is ubiquitous in the body; it is essential for pH regulation, and it provides a means for the disposal of CO_2_, a metabolic waste product. A third, emerging role is to accelerate the synthesis of cyclic nucleotides by soluble adenylate cyclase and by at least some membrane guanylate cyclases (for review, see Steegborn, 2014; Tresguerres et al., 2014; Sharma et al., 2016). ROS-GCs in the outer segments of retinal rods and cones are stimulated by bicarbonate, and given their critical role in phototransduction, bicarbonate must affect vision.

In darkness, relatively high levels of cGMP maintain cyclic nucleotide gated (CNG) ion channels in an open configuration. Influx of predominantly Na^+^ but also some Ca^2+^ through the CNG channels depolarizes the membrane to support a steady release of neurotransmitter at the synapse. The entry of Na^+^ into the outer segment is matched by the exit of K^+^ through voltage-gated channels in the inner segment. This flow of ions, known as the circulating current or the “dark” current, is blocked when photon absorption by visual pigment in the outer segment activates a biochemical cascade that leads to cGMP hydrolysis and closure of CNG channels. The resultant hyperpolarization reduces neurotransmitter release at the synapse. Response recovery depends on cascade shutoff, but ultimately requires the restoration of cGMP and the reopening of CNG channels. The basal rate of cGMP synthesis in darkness is relatively low, but during the course of the photocurrent response, ROS-GC is subject to Ca^2+^-dependent regulation. The resting [Ca^2+^]_i_ in darkness is set by an equilibrium between entry of the ion through the CNG channels and its removal by Na^+^/K^+^,Ca^2+^ exchange. CNG channel closure in response to light upsets the balance allowing continued activity of the Na^+^/K^+^, Ca^2+^ exchanger to lower [Ca^2+^]_i_. GCAPs, neuronal calcium sensing subunits of the ROS-GC complex, stimulate cGMP synthesis by ROS-GCs at low [Ca^2+^] and by doing so, restrict the size and accelerate the recovery of the electrical response to photons (for review, see Wen et al., 2014).

Bicarbonate is abundant in the retina, generated from CO_2_ by carbonic anhydrases as a by-product of the extremely high metabolic activity sustained by rods and cones, particularly in darkness (for review, see Hurley et al., 2015; Narayan et al., 2017). Bicarbonate accentuates the role of ROS-GC in phototransduction. By stimulating the synthesis of cGMP by ROS-GC in darkness, it opens a greater fraction of CNG channels to confer a larger saturated response to light (Hare and Owen, 1998; Donner et al., 1990; Koskelainen et al., 1993; Duda et al., 2015). After photon absorption, bicarbonate amplifies the Ca^2+^-mediated, negative feedback control over the photoresponse provided by ROS-GC (Duda et al., 2015), affecting response kinetics and sensitivity (Lamb et al., 1981; Baylor et al., 1984; Lamb, 1984).

The relatively low affinity of ROS-GCs for bicarbonate, with EC_50_s in the tens of mM range (Duda et al., 2015), raises the question: could gaseous CO_2_, which is always present with bicarbonate in aqueous solutions, actually be the more physiologically relevant modulator of ROS-GCs? If bicarbonate is the modulator, how does it access the ROS-GC inside the outer segments of rods and cones? The two questions are related, because CO_2_ can freely pass through membranes but as a charged molecule, bicarbonate cannot. At least some cones but not rods express carbonic anhydrase (Musser and Rosen, 1973a, b; Parthe, 1981; Nork et al., 1990) to interconvert CO_2_ and bicarbonate either to control [CO_2_]_i_ if CO_2_ is the modulator or to form bicarbonate if it is the modulator. Immunohistological localization at the outer plexiform layer of sodium dependent, bicarbonate transporters, NBCn1 (Bok et al., 2003; Boedtkjer et al., 2008) and possibly NBCe2 (Kao et al., 2011; but see Hilgen et al., 2012), provide for additional bicarbonate uptake at the synapses of photoreceptors, if bicarbonate is the modulator. Bicarbonate might also enter and exit through gap junctions or through Cl^-^ channels (Qu and Hartzell, 2000). Bicarbonate is cleared from photoreceptors by Cl^-^-dependent exchange (Koskelainen et al., 1993, 1994; Kalamkarov et al., 1996). In the present study, CO_2_ was tested as a modulator for ROS-GC in a reconstituted system and in salamander rods and cones. We further localized spatially the points of entry and egress for bicarbonate in salamander rods and cones and characterized their expression of carbonic anhydrase.

## Materials and Methods

### Animals

Larval tiger salamanders (*Ambystoma tigrinum*, Charles Sullivan and Wadelco), ∼6–10 inches in length, were kept at 12°C and fed earthworms every 10–14 d. Sex of the salamanders was not determined. All care and use conformed to the Association for Research in Vision and Ophthalmology Statement for the Use of Animals in Ophthalmic and Vision Research and to a protocol approved by the Institutional Animal Care and Use Committee. For physiologic experiments, retinas from salamanders that were dark adapted overnight were isolated under infrared illumination and stored in a MOPS-buffered Ringer’s solution on ice. In the initial experiments, the MOPS-buffered Ringer’s contained: 58 mM NaCl, 2.5 mM KCl, 1 mM MgCl_2_, 1.5 mM CaCl_2_, 5 mM HEPES, 0.02 mM EDTA, 10 mM glucose, and 55 mM MOPS; pH 7.6. The [Cl^-^] in these experiments was ∼65.5 mM. Later, the [MOPS] was reduced in some experiments with a concomitant increase in [Cl^-^] to ∼90.5 mM: 83 mM NaCl, 2.5 mM KCl, 1 mM MgCl_2_, 1.5 mM CaCl_2_, 5 mM HEPES, 0.02 mM EDTA, 10 mM glucose, and 30 mM MOPS; pH 7.6.

### Electrical recordings

After shredding a piece of retina, photocurrent responses to flashes were recorded from individual rods and cones (or from pairs of cones in a few special cases, see below) using the suction electrode technique with either outer segment (OS-in) or inner segment (IS-in) inside the pipette (Baylor et al., 1979; Makino et al., 1999). Except where expressly specified otherwise, all results are from green-sensitive rods or from red-sensitive cones. The pipette was filled with one of the MOPS-buffered Ringer’s or with a corresponding low [Cl^-^] version in which NaCl and KCl were replaced with methanesulfonate salts (Sigma); pH 7.6. Records were low pass filtered at 30 Hz with an 8-pole Bessel filter (Frequency Devices) and digitized online at 400 Hz (Patchmaster v2x53, Heka). Traces were not adjusted for the delay introduced by low pass filtering. The records shown in the figures were subjected to additional digital filtering at 8 Hz (Igor Pro v7.02, Wavemetrics, Inc.). Recordings were made at room temperature, 19–22°C.

Bicarbonate buffered Ringer’s contained 25 mM or 50 mM HCO_3_^-^ in place of an equimolar amount of MOPS; pH 7.6. Although the solutions were kept in covered reservoirs, pH sometimes changed over a time scale of hours, so pH was measured after the recording session. A working range of 7.5–7.8 was deemed acceptable. Bicarbonate, bicarbonate plus carbonic anhydrase inhibitor, or carbonic anhydrase inhibitor alone were gradually introduced to cells by bath perfusion. Circulating current changed over the subsequent 10–15 min and was typically measured 20 min after switching the perfusate. Two carbonic anhydrase inhibitors were used: acetazolamide (Sigma) and dorzolamide TCI (Tokyo Chemical Industry Company, Ltd.). Reversibility of treatment-induced changes in flash responses was generally expected except in experiments with low [Cl^-^] in the pipette, so for a given parameter, a ratio was taken of the magnitude during perfusion with bicarbonate divided by the average of the pretreatment and washed magnitudes. Cells for which parameters did not recover to within 20% of the starting value were generally not quantified. Flash response kinetics were determined for responses whose amplitudes were <0.2 of the maximum.

### Biochemical assays of cGMP synthesis

Murine carbonic anhydrase Type II and bovine ROS-GC1 cDNA were cloned into a pBudCE4.1 vector (Thermo Fisher Scientific) at the *Not*I/*Xho*I sites of the EF-1a multiple cloning site and *Sal*I/*Xba*I sites of the CMV multiple cloning site, respectively. The construct was verified by sequencing. COS cells originally purchased from ATCC (catalog #CRL-1651, RRID: CVCL_0224) and now maintained in the laboratory, were transfected with the vector using Lipofectamine (Thermo Fisher Scientific) according to the manufacturer’s protocol. Zn^2+^ at ∼0.1 g/l was present in the growth medium to enhance carbonic anhydrase activity. Seventy-two hours after transfection, the cells were placed in an incubator with humidified air containing 15% CO_2_ for 1 h. Cells were then washed with 50 mM Tris-HCl/10 mM MgCl_2_ buffer; pH 7.4, scraped into 0.5 ml of the buffer, gently homogenized and centrifuged at 3000 rpm. The amount of cGMP in the supernatant was determined by radioimmunoassay (Nambi et al., 1982). Cells were immunostained for ROS-GC1 and carbonic anhydrase II to check for co-expression of both proteins in the transfected cells, as described below.

In experiments on a core catalytic domain fragment of bovine ROS-GC1, the coding sequence for the G817-Y965 region (numbering for mature protein according to Goraczniak et al., 1994) was amplified from bovine ROS-GC1 cDNA by PCR and cloned into the *Bgl*II/*Nco*I site of pET30Xa/LIC vector (Novagen) for expression in bacterial cells (BL21 RIL). The ROS-GC1 fragment was purified first on a Ni-NTA column and then on a Superdex 200 size exclusion column by FPLC. The effect of 30 mM bicarbonate on the rate of cGMP synthesis was assessed by radioimmunoassay (Nambi et al., 1982).

### Histochemical localization of carbonic anhydrase

For confirmation of transfection, COS cells transfected with ROS-GC1 and carbonic anhydrase II cDNAs were grown on coverslips in DMEM supplemented with 10% fetal bovine serum. Seventy hours after transfection, the cells were fixed in freshly prepared 4% paraformaldehyde in PBS; pH 7.6, for 10 min at room temperature. The fixed cells were washed with Tris-buffered saline (TBS); pH 7.6, blocked in 1% preimmune donkey serum, 1% bovine serum albumin (IgG-free, protease free; Jackson ImmunoResearch Laboratories, Inc.) in TBS containing 0.5% Triton X-100 (TTBS) for 1 h at room temperature, washed with TTBS, incubated with anti-ROS-GC1 goat polyclonal antibody (1:50 dilution in blocking solution, Santa Cruz Biotechnology, Inc., catalog #376217, RRID: AB_10991113) overnight at 4°C, washed with TBS containing 0.05% Tween 20 (TBST) and then incubated with Alexa Fluor 488-conjugated donkey anti-goat antibody (1:200 dilution; Jackson ImmunoResearch Laboratories, Inc.) for 1 h at room temperature, and washed with TBST. This was followed by incubation with anti-carbonic anhydrase II, mouse, monoclonal antibody conjugated with Alexa Fluor 647 (1:40 dilution; Santa Cruz Biotechnology, Inc., catalog #sc-48351, RRID: AB_626796). After a final wash with TBS, the cells were examined under a confocal microscope using excitation and emission wavelengths of 493 and 519 nm, respectively, for Alexa Fluor 488 and 651 and 667 nm, for Alexa Fluor 647.

For labeling of retina, salamanders were dark adapted for ∼30 min. After euthanasia, eyecups were isolated in dim ambient lighting and fixed in freshly prepared 4% paraformaldehyde in TBS at 4°C for 1 h, cryoprotected in 30% sucrose at 4°C, mounted in O.C.T. solution (Electron Microscopy Sciences), frozen at –80°C, and cut into 25-µm sections using a Hacker-Bright OTF5000 microtome cryostat (Hacker Instruments and Industries Inc.). Sections were washed four times with PBS, incubated in 100% methanol at –20°C for 5 h and washed four to five times with PBS. Washed sections were incubated with rhodamine labeled peanut agglutinin antibody (Vector Laboratories, catalog #RL-1072, RRID: AB_2336642) in TBS containing 1% Triton X-100 for 1 h at room temperature, washed several times with TBST, and incubated overnight with the anti-carbonic anhydrase II mouse antibody (Santa Cruz Biotechnology, Inc.) in TTBS containing 1% bovine serum albumin at 4°C, washed four to six times with TBST, incubated with Alexa Fluor 488-conjugated secondary antibody (Jackson ImmunoResearch Laboratories) and washed with TBS. Rhodamine was excited with 550–555 nm light and emission was detected at 575–580 nm; for Alexa Fluor, excitation and emission wavelengths were 493 and 519 nm, respectively.

Images were acquired at 22°C using an inverted Olympus IX81 Microscope/FV1000 Spectral Laser Confocal System outfitted with a 60x objective PLAPON 60X OTIRFM, numerical aperture = 1.45, and analyzed using Olympus FluoView FV10-ASW v01.07 software (Olympus Corp.). Digital images were processed using Photoshop Elements 6 software (Adobe Systems, Inc.).

### Experimental design and statistical analyses

Statistical evaluations of the effect of cell type, recording configuration and bicarbonate concentration on physiologic parameters were made with a Kruskal–Wallis nonparametric test (Stata v11.2, StataCorp LLC) and Dunn’s test for *post hoc* comparisons (Dinno, 2015). A repeated measures linear regression with circulating current as the dependent measure was performed with XTMIXED of Stata to take into account multiple measurements on the same cell, testing separately, the treatments applied to rods and cones; *p* ≤ 0.05 was considered to be significant. Curve fitting to determine relative sensitivity to flashes was conducted using Igor Pro.

Biochemical assays to test for the effect of CO_2_ were performed in triplicate and repeated three times. The effect of different conditions on the cGMP accumulation in COS cells, relative to that in COS cells expressing ROS-GC1 in air for each assay, was evaluated by an ANOVA with subsequent Bonferroni *post hoc* testing (Stata).

Biochemical assays on the core catalytic domain fragment were performed in triplicate and repeated twice. The effect of bicarbonate was assessed by a *t* test without assuming equal variances (Stata).

## Results

### Bicarbonate sensing by ROS-GC

**Figure 1.**
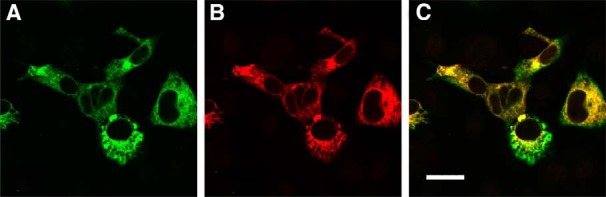
Immunohistochemical confirmation of ROS-GC1 and carbonic anhydrase II co-expression in COS cell cultures. ***A***, Anti-ROS-GC1. ***B***, Anti-carbonic anhydrase II. ***C***, Composite. Scale bar, 30 μm.

**Figure 2.**
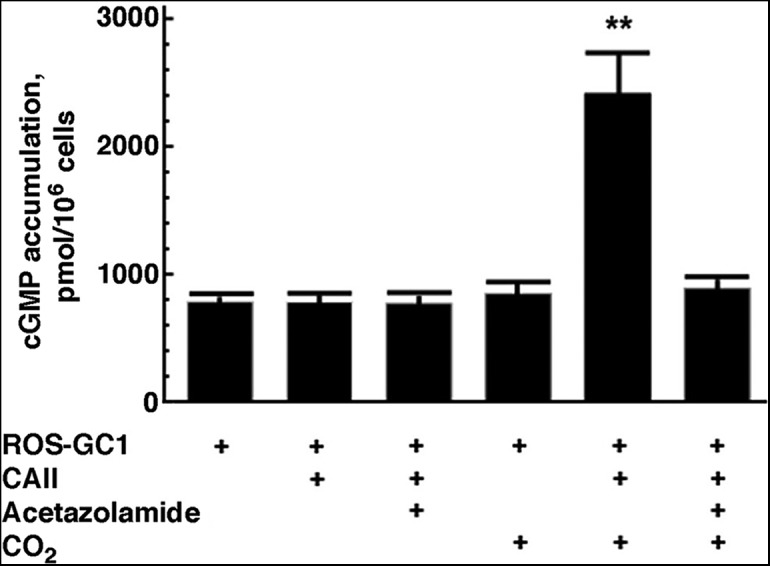
CO_2_-induced stimulation of cGMP accumulation in COS cells co-expressing bovine ROS-GC1 and murine carbonic anhydrase II. Co-expression of carbonic anhydrase II with or without 80 µM acetazolamide had no effect on cGMP accumulation in cells incubated in air for 1 h. But when the assay was conducted in a 15% CO_2_ atmosphere, cGMP synthesis was much higher in the cells co-expressing carbonic anhydrase II. Asterisks indicate a difference (*p* < 0.0005) from every other condition, based on an ANOVA, *F*_(5,12)_ = 79.83 (*p* < 0.00005) and a Bonferroni *post hoc* test. CO_2_ had no effect on cells lacking carbonic anhydrase II expression and the invigorating effect of CO_2_ on cells with carbonic anhydrase II was blocked by 80 µM acetazolamide. Error bars demarcate SEM, *n* = 3.

Tens of mM bicarbonate are required to increase production of cGMP in biochemical assays of ROS-GC1 catalytic activity (Duda et al., 2015), calling into question the identity of its true modulator; is it indeed bicarbonate or rather its precursor, CO_2_? Perhaps ROS-GC1 responds to a much lower amount of CO_2_ that exists in equilibrium with the bicarbonate in aqueous solution. As a test, COS cells were co-transfected with bovine ROS-GC1 and murine carbonic anhydrase II and assayed for cGMP synthetic activity in the presence of CO_2_. Co-expression of the two enzymes was verified immunohistochemically (Fig. 1). A representative experiment, repeated in triplicate, is shown in Figure 2. From four such experiments (ANOVA, *F*_(7,16)_ = 15.91, *p* < 0.00005), cGMP accumulation increased 3.5 ± 0.5-fold (mean ± SEM) in the cells co-expressing ROS-GC1 and carbonic anhydrase II when they were placed in a high CO_2_ atmosphere, compared to cells expressing ROS-GC1 alone and tested in air (Bonferroni *post hoc* test, *p* < 0.002). The effect was blocked by carbonic anhydrase inhibitors; in the presence of 80 μM acetazolamide, cGMP accumulation was only 30 ± 10% (*n* = 3) above the cGMP levels in cells incubated in the air only, and with 200 μM dorzolamide, it was only 10% (*n* = 1) higher. In preliminary experiments, a lower, 50 μM concentration of acetazolamide was less effective in blocking cGMP accumulation. Cyclic GMP levels in COS cells expressing ROS-GC1 alone increased non-significantly by 13 ± 2% (*n* = 4) in the presence of high CO_2_. There were no significant effects in other control experiments conducted in air with either carbonic anhydrase co-expression or with carbonic anhydrase plus carbonic anhydrase inhibitor; the changes in levels were only +2 ± 1% (*n* = 4), –1 ± 1% with acetazolamide (*n* = 3), and +1% with dorzolamide (*n* = 1), respectively. Since the increases in guanylate cyclase activity in cells co-expressing ROS-GC and carbonic anhydrase on exposure to CO_2_ were similar to those observed with membranes of transfected COS cells or with retinal membranes (Duda et al., 2015) challenged with bicarbonate, we conclude that ROS-GC was targeted directly by bicarbonate and that gaseous CO_2_ was its source.

To localize the interaction between bicarbonate and ROS-GC1, a soluble fragment consisting of the core catalytic domain from G817-Y965 was expressed in bacterial cells and purified. The fragment formed a homodimer in solution, as verified by molecular weight markers during FPLC elution and gel electrophoresis performed without reducing agents. In accordance with its biochemical feature that in the absence of its fusion with the plasma membrane it retains reduced catalytic activity (Ravichandran et al., 2017), the fragment exhibited a low, basal activity of 9.0 ± 0.5 pmol cGMP/min/mg prot (*n* = 2) that increased to 31.0 ± 1.6 pmol cGMP/min/mg prot (*n* = 2) when 30 mM bicarbonate was present (two tailed *t* test with unequal variances, *t*_(1.58)_ = –13.12, *p* = 0.0133). These experiments demonstrated direct bicarbonate binding to and stimulation of the core catalytic domain of ROS-GC1, as is the case for ONE-GC (Duda and Sharma, 2010), and ruled out the need for an intermediary protein.

### Entry and exit of bicarbonate in rods

**Figure 3.**
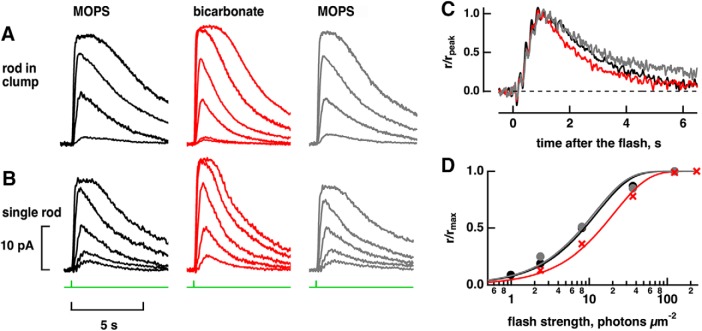
Increased circulating current, faster flash response kinetics, and reduced sensitivity in rods recorded with OS-in on treatment with 50 mM bicarbonate. ***A***, Rod attached to a clump consisting of a cone and five other cells. Flash strengths were: 0.32, 4.8, 17.1, and 72.4 photons μm^−2^ at 520 nm in MOPS (left), 0.32, 0.59, 4.8, 17.1, 72.4, and 258 photons μm^−2^ at 520 nm in bicarbonate (middle) and 0.59, 4.8, 17.1, and 72.4 photons μm^−2^ at 520 nm in the MOPS wash (right). A brighter flash of 258 photons μm^−2^ was necessary to obtain the saturating response during bicarbonate treatment (middle panel). ***B***, Isolated rod that retained its spherule. Flash strengths were: 1.0, 2.4, 8.1, 36.2, and 124 photons μm^−2^ at 500 nm in MOPS (left, right) and 2.4, 8.1, 36.2, 124, and 239 photons μm^−2^ at 500 nm in bicarbonate (middle). ***C***, Faster flash response recovery with bicarbonate for the isolated rod in ***B***. Dim flash responses, whose peak amplitudes were less than a fifth of the maximum, were scaled to their peak amplitudes. Integration time, given as the integrated area of the normalized dim flash response, decreased ∼16% with bicarbonate. ***D***, Loss in sensitivity to flashes of the rod in ***B*** with bicarbonate. Results were fit with a saturating exponential function: r/r_max_ = 1 – exp(-ki) where k is a constant equal to ln(2)/i_0.5_ and i_0.5_ is the flash strength eliciting a half-maximal response.

Given that bicarbonate is the modulator for ROS-GC and that it does not easily cross membranes, the points of entry for bicarbonate into photoreceptors were determined by mechanically dissociating retinas and making electrical recordings with either the outer segment (OS-in) or inner segment (IS-in) of a rod or cone pulled inside a tight-fitting, glass pipette. With OS-in recording, bicarbonate in the bath was accessible only to the inner segment and vice versa for IS-in recordings. The amplitude of the saturating flash response provided a measure of the circulating current in darkness. Addition of 50 mM bicarbonate to the bath increased the circulating current of four green-sensitive rods attached to large pieces of retina in OS-in recordings by 15 ± 3%, similar to previous reports (Lamb et al., 1981; Duda et al., 2015). Large pieces of retina contained many other rods and cones along with inner retinal neurons and Müller glia. Isolated rods, recorded with OS-in, do not typically respond to bicarbonate (Duda et al., 2015) or bicarbonate plus acetazolamide (Donner et al., 1990). To find out whether Müller cells were needed for uptake of bicarbonate into rods, two rods attached to small clumps of one to six other cells were recorded with OS-in. A cone was included in both clumps; intact Müller cells were not present in either clump. Circulating current was enlarged by 50 mM bicarbonate (Fig. 3*A*), and flash response kinetics were faster, indicating that bicarbonate uptake was preserved in clumps. We conclude that bicarbonate uptake by rods did not depend on contact with or close proximity to Müller cells.

**Figure 4.**
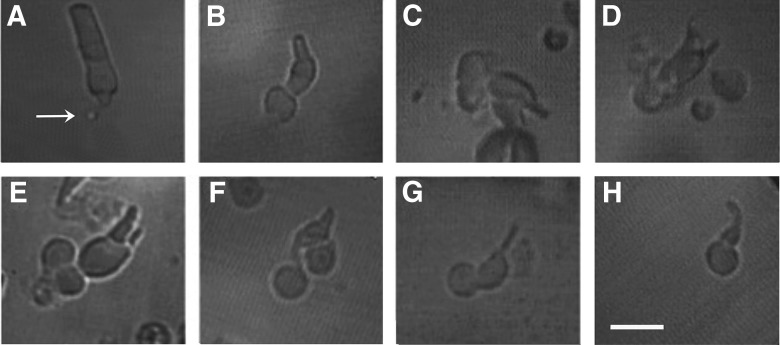
Isolated rods and cones. ***A***, Green-sensitive rod with spherule (arrow). ***B***, Large, single, red-sensitive cone. ***C***, Twin cone with the outer segment of one member in the pipette. ***D***, Same cone after ejection from the pipette. ***E***, Double cone. ***F***, “Chief” member of a double cone with thicker outer segment. A remnant of its accessory cone is still attached on the right side. ***G***, “Accessory” member of a double cone with thinner outer segment. Images were acquired under infrared light. ***H***, Single, UV-sensitive cone. Scale bar, 20 μm.

Reasoning that access to bicarbonate must have required the synaptic region or spherule, which was intact for rods in large pieces of retina and in small clumps of cells but was usually broken off during the mechanical dissociation, we sought rare, isolated rods that retained their spherules (Fig. 4*A*). Although many more rods retain their spherule when dissociated with papain (Bader et al., 1978), we wished to avoid any complications arising from the enzymatic digestion of phototransduction cascade components (Aton and Litman, 1984). With OS-in, four out of four isolated rods with spherule indeed responded reversibly to bicarbonate with a circulating current that increased by 17 ± 3%, an integration time of the dim flash response that decreased by 21 ± 4% (*n* = 3) indicating faster flash response recovery, and a sensitivity to flashes that was reduced by 52 ± 10% (*n* = 2; Fig. 3*B–D*).

**Figure 5.**
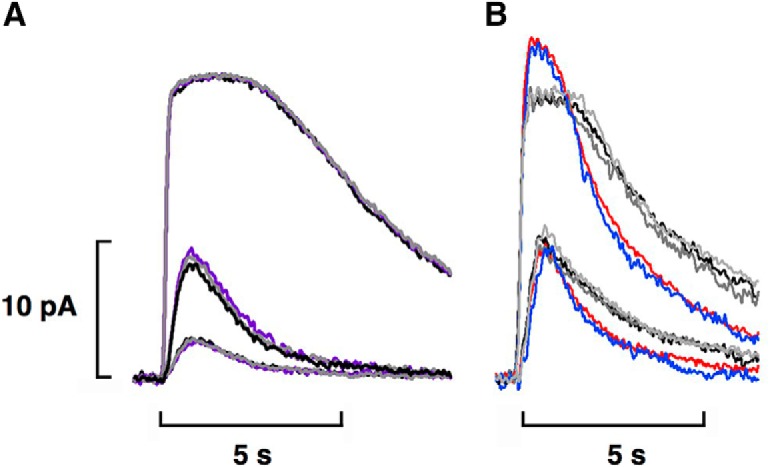
Lack of effect of carbonic anhydrase inhibitors on phototransduction in rods. ***A***, OS-in recording of an isolated rod lacking a spherule. Averaged responses to flashes of 1.1, 5.2, and 154 photons μm^−2^ at 500 nm: pretreatment, black; during perfusion of the inner segment with 250 μM acetazolamide, violet; after extensive washing to remove the acetazolamide, gray. ***B***, OS-in recording from an isolated rod with spherule of averaged responses to flashes of 8.1 photons μm^−2^ that were half-saturating in MOPS and to saturating flashes of 124 photons μm^−2^ at 500 nm: pretreatment, black; 50 mM bicarbonate, red; wash, gray; 32 μM acetazolamide + 50 mM bicarbonate, blue; final wash, light gray.

In control experiments on 15 rods recorded with OS-in (Fig. 5*A*) and three rods recorded with IS-in, 2 µM to 250 µM acetazolamide or 500 µM dorzolamide alone did not significantly change circulating current (+1 ± 2%, *n* = 18), integration time (+1 ± 4%, *n* = 12), or sensitivity (+7 ± 2%, *n* = 11). The lack of effect of carbonic anhydrase inhibitor on five of these OS-in rods that were attached to a clump or to large pieces of retina meant that under our experimental conditions, rods did not take up appreciable quantities of bicarbonate produced endogenously by cones or Müller cells or any other retinal cells. In other control experiments, two isolated rods treated with bicarbonate plus acetazolamide at concentrations up to 32 µM (Fig. 5*B*) and one rod treated with bicarbonate plus 100 μM dorzolamide exhibited changes in circulating current similar to those obtained for treatment with bicarbonate alone. The results of both control experiments were consistent with the absence of an effect of carbonic anhydrase inhibitor on the phototransduction apparatus of rods (Donner et al., 1990).

**Figure 6.**
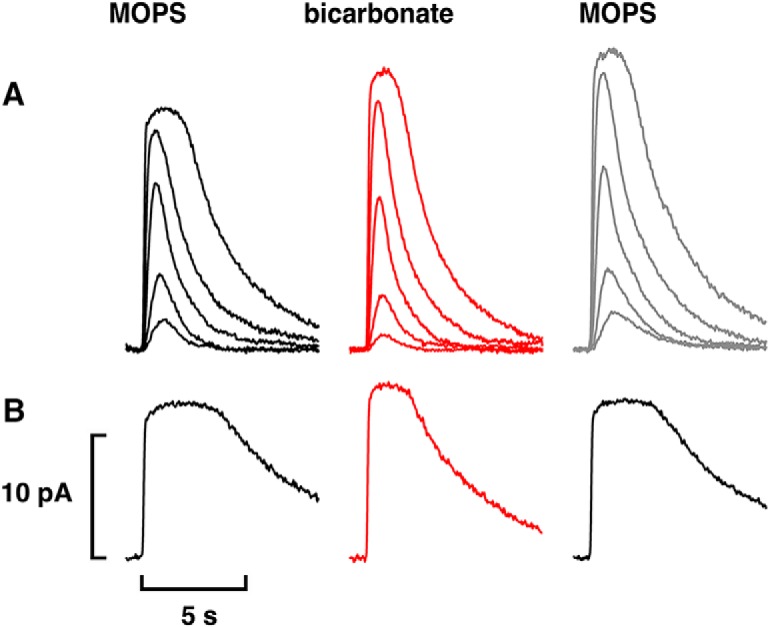
Cl^-^ dependence of bicarbonate efflux from rods. Averaged responses of two rods to flashes while recording OS-in with low [Cl^-^] in the pipette. Circulating current increased on bath perfusion with 25 mM bicarbonate. ***A***, Circulating current in one rod continued to increase during washing with MOPS Ringer’s because internal bicarbonate was denied egress. Flash strengths at 500 nm were: 0.5, 1.8, 8.2, 28.0, and 125 photons μm^−2^. ***B***, Partial ejection of a different rod from the pipette to allow access of its OS to higher [Cl^-^] during the wash restored the prolonged time in saturation of the bright flash response and enabled the circulating current to subside to pre-treatment levels. The OS was pulled back into the pipette for recording after the OS wash. Flash strength at 500 nm was 223 photons μm^−2^.

Post-treatment washing of the rod with MOPS buffered Ringer’s reversed the effects of bicarbonate (Figs. 3, 5*B*). An HCO_3_^-^/Cl^-^ exchanger in the outer segment couples the entry of Cl^-^ to the extrusion of internal bicarbonate (Koskelainen et al., 1994; Kalamkarov et al., 1996). Here we show that filling the pipette with a low Cl^-^ Ringer’s solution in OS-in recordings to suppress the HCO_3_^-^/Cl^-^ exchanger trapped bicarbonate inside the rod and prevented circulating current from subsiding to the pretreatment level after washing (Fig. 6*A*). Circulating current even increased during the time it took to clear the bath of bicarbonate. However, if a portion of the OS remained outside of the electrode allowing access to the higher Cl^-^ in the bath during the wash (*n* = 2) or if the rod OS was partially ejected for washout and then pulled back into the pipette for recording (*n* = 2), then the effect of bicarbonate on circulating current was fully reversible (Fig. 6*B*). It was typically difficult to detect changes with 25 mM bicarbonate but with OS-in and low Cl^-^ in the pipette, this treatment increased circulating current by 8 ± 1% (*n* = 5), shortened integration time of the dim flash response by 22 ± 5% (*n* = 4) and reduced sensitivity by 34 ± 12% (*n* = 3, not significant) with recovery only after exposing the outer segment to high Cl^-^ in the bath. All rods in these experiments were attached to large pieces or clumps or if isolated, retained a synapse. We conclude that extrusion by an HCO_3_^-^/Cl^-^ exchanger located at the base of the OS, if not along its entire length, was the principal means for removing bicarbonate from the rod.

### Access to bicarbonate in cones

**Figure 7.**
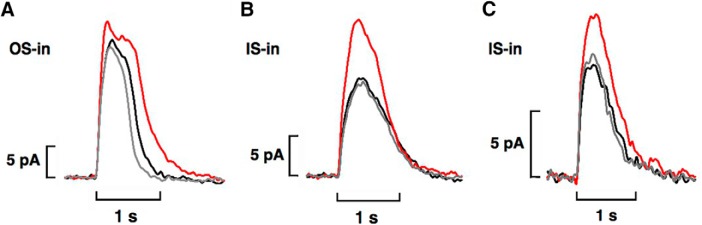
Increase in circulating current with bicarbonate on two single cones recorded with OS-in (***A***) or with IS-in (***B***) and one double cone with IS-in for both cones (***C***). Traces obtained with IS-in have been inverted for ease of comparison with OS-in recordings: pretreatment, black; during treatment with 50 mM bicarbonate (***A***, ***B***) or with 25 mM bicarbonate (***C***), red; after washing, gray. Flashes at 600 nm were 62,500 photons μm^−2^ (***A***), 33,900 photons μm^−2^ (***B***), and 43,500 photons μm^−2^ (***C***).

Isolated cones or cones in a clump responded to 50 mM bicarbonate with an increase in circulating current in OS-in recordings (Fig. 7*A*), consistent with previous studies (Koskelainen et al., 1993; Duda et al., 2015). In the Duda et al. (2015) study, all cones were red-sensitive, single cones (Fig. 4*B*). Here, we extend observations to twin (Fig. 4*C*,*D*) and to double (Fig. 4*E–G*) cones, which are also red-sensitive (Attwell et al., 1984; Isayama et al., 2014) and to UV-sensitive cones (Fig. 4*H*). Twin cones consist of two members that are symmetric in morphology, whereas the two members in double cones differ in the sizes and shapes of their inner and outer segments (Walls, 1963). Usually only one member of a double or twin cone was recorded but there were a few recordings with both members inside the pipette. While salamander double cones are not electrically coupled (Attwell et al., 1984), the situation for twin cones is not known. For two double and five single red-sensitive cones, current increased 1.32 ± 0.07-fold with 50 mM bicarbonate and for three UV-sensitive cones, it increased 1.18 ± 0.08-fold. On average, the impact of bicarbonate on red-sensitive cones was greater than that on rods (*p* = 0.032).

Rods lacking a spherule (synaptic zone) and recorded with IS-in did not respond to 50 mM bicarbonate (*n* = 4), as reported in Duda et al. (2015). However, the circulating current increased in 18 red-sensitive cones with IS-in in the present study, including two double cones (Fig. 7*B*,*C*). In three of the cones, bicarbonate was applied a second time after washing with the same result. The relative increase in current brought about by bicarbonate was as large, if not larger, in cones with IS-in (1.45 ± 0.05-fold) than with OS-in (Fig. 7*A*,*B*), although the difference did not quite reach significance (*p* = 0.11). Halving the concentration of bicarbonate from 50 to 25 mM produced a smaller, 1.22 ± 0.08-fold (*n* = 7, *p* = 0.015) effect in IS-in recordings (Fig. 7*B*,*C*). The comparisons were made with a rank order test (Kruskal–Wallis, χ^2^ = 14.08, 3 df, *p* = 0.0028) followed by Dunn’s *post hoc* testing. The circulating current in one cone increased from 7 pA in MOPS to 12 pA in 50 mM bicarbonate, then diminished to 9 pA in 25 mM bicarbonate and returned to 12 pA in 50 mM bicarbonate, providing further support that the lower concentration of bicarbonate was less effective.

**Figure 8.**
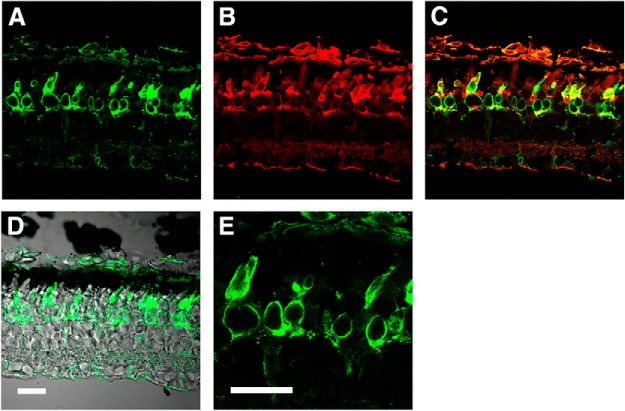
Expression of carbonic anhydrase II in salamander cones. Cones were labeled with anti- carbonic anhydrase II antibody (***A***) as well as peanut agglutinin conjugated with rhodamine (***B***). ***C***, Composite. ***D***, Overlay of carbonic anhydrase staining onto DIC image. ***E***, Higher magnification of a double cone (far left) and a single cone (far right) from ***A***. Scale bar in ***D*** applies to panels ***A–D***. Scale bars, 30 μm.

The presence of carbonic anhydrase activity in some vertebrate cones (Musser and Rosen, 1973a, b; Parthe, 1981; Nork et al., 1990) suggests that CO_2_ in equilibrium with exogenously applied bicarbonate could cross the plasma membrane and generate bicarbonate internally. To find out whether salamander photoreceptors express carbonic anhydrase, retinas were probed with peanut agglutinin lectin, which binds with high affinity to the saccharides in the cone extracellular matrix (Blanks and Johnson, 1984) and with an antibody raised against carbonic anhydrase II. Carbonic anhydrase II antibody and peanut agglutinin binding co-localized, verifying the carbonic anhydrase II expression in salamander cones (Fig. 8). Labeling was pervasive across the cones, inclusive of double and single cones, but was absent from rods.

**Figure 9.**
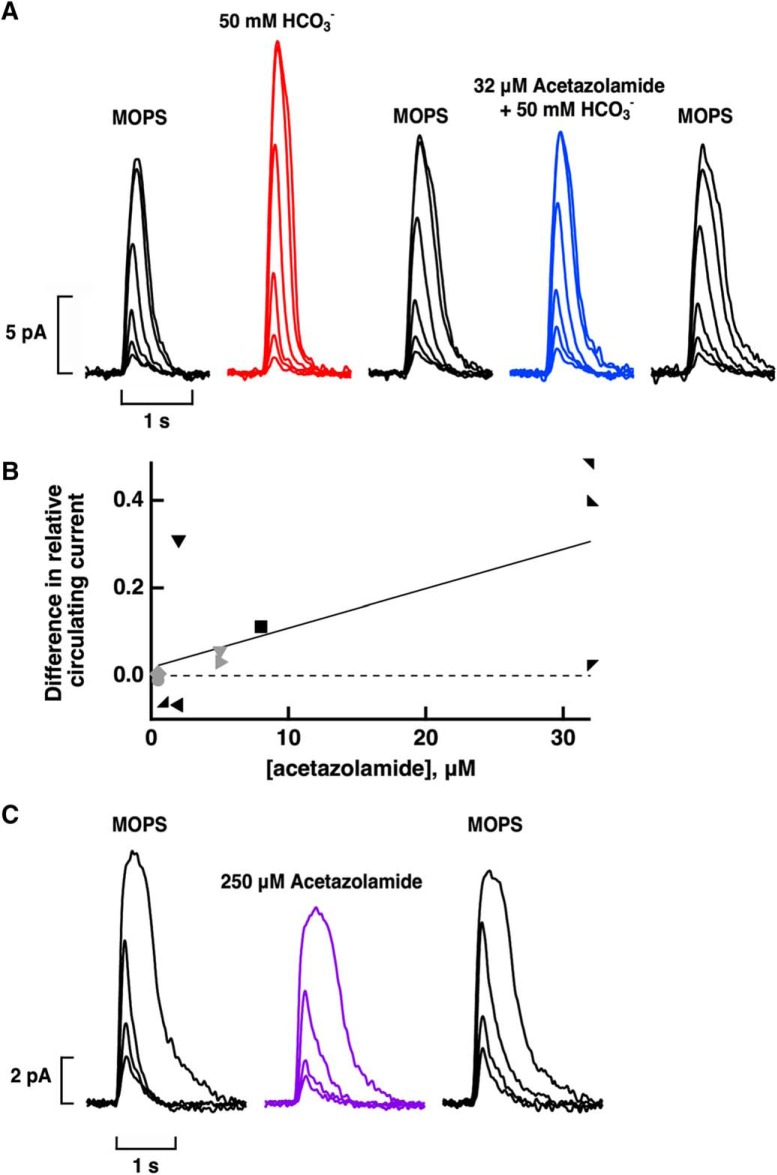
Reduced circulating current in the presence of the carbonic anhydrase inhibitor acetazolamide in cones. ***A***, Acetazolamide blockade of the bicarbonate effect. Flash strengths were: 321, 597, 1300, 4530, 17,900, and 33,300 photons μm^−2^ at 600 nm. Recorded with IS-in, but traces are inverted for ease of comparison with OS-in recordings. ***B***, Dose-response relations for acetazolamide. Difference is given by the relative circulating current with 50 mM bicarbonate minus the relative circulating current with 50 mM bicarbonate plus acetazolamide for 11 cones subjected to both treatments. IS-in, black symbols; OS-in, gray symbols. Continuous line from linear regression has an *r* ^2^ value = 0.41, *p* = 0.033 for the slope. Dashed line shows the result that would be obtained if acetazolamide were to have had no effect. ***C***, Reduced circulating current in an isolated cone with OS-in on treatment with acetazolamide alone. Flash strengths were: 246, 459, 1860, and 25,600 photons μm^−2^ at 600 nm.

Next, the internal conversion of CO_2_ to bicarbonate in salamander cone OSs was tested by co-application of bicarbonate with a carbonic anhydrase inhibitor, acetazolamide or dorzolamide. Dorzolamide at concentrations of 100–500 μM failed to block or even diminish the increase in circulating current with bicarbonate in cones recorded with IS-in (*n* = 5). Acetazolamide at concentrations as low as 1 μM did prevent bicarbonate from increasing the circulating current in some cells (Fig. 9*A*), however, a reduced increase in current persisted in most cells. On analysis with a linear model with mixed effects (Wald χ^2^ = 48.12, 65 observations, *p* < 0.0005), the overall effect on circulating current of 50 mM bicarbonate plus acetazolamide at concentrations ranging from 1 to 160 μM was an increase of 28 ± 5% (*n* = 17), less than the above 45% increase seen with bicarbonate alone on cones recorded with IS-in (*p* = 0.002). In a subset of seven cones recorded IS-in and four cones recorded OS-in that were treated sequentially with bicarbonate and then with bicarbonate plus acetazolamide, increasing the concentration of acetazolamide improved its efficacy in reducing the effect of bicarbonate (Fig. 9*B*). Internal conversion of CO_2_ to bicarbonate therefore contributed to the modulation of circulating current in cones.

In three out of three isolated cones recorded OS-in, treatment with 250 μM acetazolamide but no added bicarbonate reduced the circulating current by 23 ± 2% (Fig. 9*C*). Chloride concentration was 90.5 mM inside the electrode and in the bath for these experiments. Apparently, acetazolamide blocked the ongoing synthesis of bicarbonate from endogenous CO_2_ that was otherwise high enough to stimulate ROS-GC activity in the cone outer segment.

There were no cases in which the circulating current during treatment with bicarbonate and carbonic anhydrase inhibitor fell below the pretreatment value. Therefore, some bicarbonate must have gained entry into cone OSs. One possibility may have been via reversed operation of a bicarbonate exchanger. The bicarbonate exchanger in cones imports bicarbonate when the retina is perfused with 6 mM bicarbonate in a low Cl^-^ Ringer’s solution to create an inward driving force on bicarbonate and an outward driving force on Cl^-^ (Koskelainen et al., 1993). In our IS-in recordings, cones were treated with 25 mM rather than 50 mM bicarbonate to lower the inward driving force on bicarbonate and [Cl^-^] in the pipette was lowered to ∼5 mM to reduce [Cl^-^] inside the cone. The [Cl^-^] in the bath remained high to set up an inward driving force on Cl^-^ at the outer segment. Nevertheless, the bicarbonate-induced increase in circulating current (19 ± 5%, *n* = 11) was unaffected by the low [Cl^-^] in the pipette. Experiments conducted in which [Cl^-^] in the bath was increased from ∼65.5 to ∼90.5 mM to make reversed bicarbonate exchange even less favorable did not seem to make a difference, nor did the addition of dorzolamide to the bath. Perhaps 25 mM bicarbonate in the bath was still high enough to overcome the Cl^-^ gradient and allow a net inward movement of bicarbonate by the exchanger.

### Potency of carbonic anhydrase inhibitors

**Figure 10.**
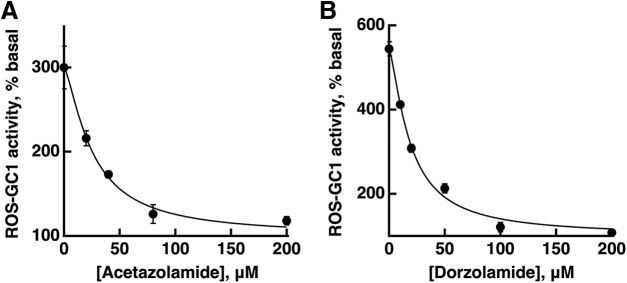
Low sensitivity of COS cells transfected with ROS-GC1 and ROS-GC1-CAII cDNAs to carbonic anhydrase inhibitors. Approximately 70 h after transfection, the cells were incubated with the indicated concentrations of acetazolamide (***A***), or dorzolamide HCl (***B***), in the presence of 15% CO_2_. The amount of cGMP formed determined by radioimmunoassay served as a measure of ROS-GC1 activity. Each graph plots the results of one experiment conducted in triplicate. In the absence of CO_2_, the amount of cGMP formed (basal ROS-GC1 activity) was 190 pmol cGMP/mg protein for the experiment in ***A***, and 160 pmol cGMP/mg protein for the experiment in ***B***. Results were fit with: activity = (maximal activity - basal activity)/(1 + ([inhibitor]/IC_50_)^n^) + basal activity. For acetazolamide, IC_50_ was 25 μM, *n* = 1.4 whereas for dorzolamide, IC_50_ was 19 μM, *n* = 1.4.

Acetazolamide was used in our early physiologic recordings at concentrations as low as 0.5 μM, because 5 μM blocked all carbonic anhydrase activity in histologic studies on retina from a number of species (Musser and Rosen, 1973a). The increased effectiveness of acetazolamide over tens of μM in reducing the bicarbonate-induced increase in current in cones of the present study and the finding in our cell-based assays for cGMP synthesis, that 50 μM acetazolamide failed to fully inhibit CO_2_ stimulated, ROS-GC activity were surprising. To investigate further, we determined the dose-response relations to characterize the effectiveness of acetazolamide and dorzolamide in blocking the production of bicarbonate by carbonic anhydrase II expressed in COS cells that also expressed recombinant ROS-GC1. One experiment with each inhibitor is shown in Figure 10. In control experiments, ROS-GC1 activity was unaffected by inhibitors in the absence of CO_2_, with or without co-expression of carbonic anhydrase II (Fig. 2). Under the conditions of our experiments with COS cells, the IC_50_ for acetazolamide was 37 ± 12 μM, *n* = 2 experiments. For dorzolamide, IC_50_ was 20 ± 3 μM, *n* = 3 experiments.

## Discussion

### Bicarbonate directly stimulates ROS-GC

In *Caenorhabditis elegans*, the membrane guanylate cyclase GY-9 functions as a CO_2_ detector (Smith et al., 2013). The hypothesis that ROS-GC1 responds preferentially to CO_2_ was ruled out in biochemical assays using a recombinant system. While the possibility that CO_2_ had a marginal capacity to bind and stimulate ROS-GC1 cannot be ruled out, any slight increases in cGMP accumulation in ROS-GC1 expressing COS cells evoked by high CO_2_ were most probably due to the spontaneous formation of bicarbonate that approached an equilibrium concentration of 4 mM, by the Henderson–Hasselbalch equation. Cells co-expressing carbonic anhydrase formed much more cGMP, consistent with Duda et al. (2018), except when a carbonic anhydrase inhibitor was present (Fig. 2) indicating that ROS-GC1 responded primarily to bicarbonate. Unexpectedly, dose-response determinations for COS cells (Fig. 10) yielded IC_50_ values for acetazolamide and dorzolamide that were considerably higher than those for cell free preparations (for review, see Sugrue, 1996; Supuran and Scozzafava, 2007).

It may be argued that bicarbonate did not interact with ROS-GC1 directly, but instead bound to an accessory protein that then modulated the cyclase's catalytic activity. Mutagenesis experiments on an olfactory guanylate cyclase, ONE-GC, localized stimulation of enzymatic activity by bicarbonate to its core catalytic domain (Sun et al., 2009; Duda and Sharma, 2010), a segment that is highly conserved across membrane guanylate cyclases. We therefore expressed and purified a soluble fragment of ROS-GC1 consisting of the core catalytic domain and tested it for sensitivity to bicarbonate. The fragment formed a dimer with a relatively low basal rate of cGMP synthetic activity, but activity increased 3.4-fold with 30 mM bicarbonate. We conclude that bicarbonate binds directly to the core catalytic domain of ROS-GC1 to stimulate its enzymatic activity and that an accessory protein is not required.

### Points of bicarbonate ingress and egress in rods

By varying the extent of mechanical dissociation of the retina in conjunction with suction electrode recording to restrict bicarbonate access to specific subcellular regions of rods, we localized bicarbonate uptake exclusively to the synapse. Potential pathways for entry at the spherule include electroneutral sodium bicarbonate co-transport by NBC3, also known as NBCn1 (Bok et al., 2003; Boedtkjer et al., 2008), electrogenic sodium bicarbonate co-transport by NBCe2 (Kao et al., 2011; but cf. Hilgen et al., 2012) and permeation through Ca^2+^ activated Cl^-^ channels (Qu and Hartzell, 2000; MacLeish and Nurse, 2007). Ca^2+^ activated Cl^-^ channels are opened by depolarization and by Ca^2+^, conditions favored in darkness. The identity of the channel(s) has yet to be clarified because immunolabeling for TMEM16A and TMEM16B yielded conflicting results (Yang et al., 2008; Stöhr et al., 2009; Mercer et al., 2011; Dauner et al., 2013; Jeon et al., 2013; Caputo et al., 2015). Our results exclude entry of bicarbonate into isolated rods through hemi-gap-junction channels in peri-nuclear regions of the inner segment (Custer, 1973; Zhang and Wu, 2004) under our experimental conditions.

Internalized bicarbonate is removed by HCO_3_^-^/Cl^-^ exchange at the OS because 4-4’-diisothiocyanatostilbene-2,2’-disulphonic acid (DIDS), an agent that blocks Na^+^-independent bicarbonate transport, increases the circulating current in rods being exposed to bicarbonate. High [bicarbonate] and low [Cl^-^] around the OS actually cause rods to import bicarbonate by reversed action of the exchanger (Koskelainen et al., 1994; Duda et al., 2015). In the present study, bicarbonate was trapped inside rods for tens of minutes by lowering [Cl^-^] around the OS (Fig. 6) indicating that HCO_3_^-^/Cl^-^ exchange in the OS was the principal means for clearing bicarbonate from the rod. Egress of bicarbonate through Cl^-^ channels at the spherule or through hemi-gap-junction channels may have been very slow and reversed action of the Na^+^-dependent transporter may have been stymied by the low [Na^+^]_i_. The restricted location for egress at the outer segment ensures that bicarbonate would distribute throughout the cell and importantly, accumulate in a location where it would impact visual transduction by stimulating ROS-GC.

### Additional pathways for accessing bicarbonate in cones

Cones, like rods, express Ca^2+^-activated Cl^-^ channels (Maricq and Korenbrot, 1988; Barnes and Hille, 1989; Mercer et al., 2011) and presumably express transporters at the synaptic zone. Unlike most rods, mechanically dissociated cones retain the synaptic zone and take up bicarbonate when recorded with OS-in (Fig. 7*A*). Larger effects were obtained with a lower (6 mM) dose of bicarbonate in ERG recordings (Donner et al., 1990; Koskelainen et al., 1993), perhaps because inner and outer segment pathways for uptake were invoked and removal was minimized.

Carbonic anhydrase activity is present in cones of several species (Musser and Rosen, 1973a, b; Parthe, 1981; Nork et al., 1990). The majority of human cones, probably red- and green-sensitive but not blue-sensitive cones, express carbonic anhydrase activity (Wistrand et al., 1986; Nork et al., 1990). Here, we show carbonic anhydrase II immunoreactivity in single and double red-sensitive cones of salamander (Fig. 8) but remain uncertain about the blue-sensitive cones because they comprise such a minor fraction of the photoreceptor population (Sherry et al., 1998; Chen et al., 2008). Conversion of endogenous CO_2_ to bicarbonate inside cones was sufficient to increase ROS-GC activity in the outer segment, because circulating current was reduced by the addition of acetazolamide without added bicarbonate (Fig. 9*C*). While inhibition of carbonic anhydrase with acetazolamide prevented exogenously applied bicarbonate from increasing circulating current in some cones (Fig. 9*A*), block was incomplete in most cones (Fig. 9*B*; see also Koskelainen et al., 1993) even for acetazolamide concentrations as high as 160 μM. Photoreceptors are not known to express other carbonic anhydrases. Carbonic anhydrase XIV, which is included along with carbonic anhydrase II in some photoreceptor proteomes (Liu et al., 2007; Kwok et al., 2008; Skiba et al., 2013), may be a contaminant of Müller cells in which it is highly expressed (Nagelhus et al., 2005; Ochrietor et al., 2005). Regardless, acetazolamide is an effective inhibitor of both isoforms (for review, see Supuran and Scozzafava, 2007). The smaller increase in bicarbonate induced circulating current with acetazolamide in IS-in recordings meant that cones produced bicarbonate internally from CO_2_ in equilibrium with bicarbonate in aqueous solution. These results help to account for the reduced photopic a-wave of the mammalian ERG particularly with bright flashes on administration of carbonic anhydrase inhibitor (Broeders et al., 1988; Odom et al., 1994; Sauvé et al., 2006) and indicate that it was not just due to acidosis. Dorzolamide inhibited recombinant murine carbonic anhydrase II in COS cells (Fig. 10*B*), but for reasons that are not known, it was ineffectual when applied to salamander cones.

**Figure 11.**
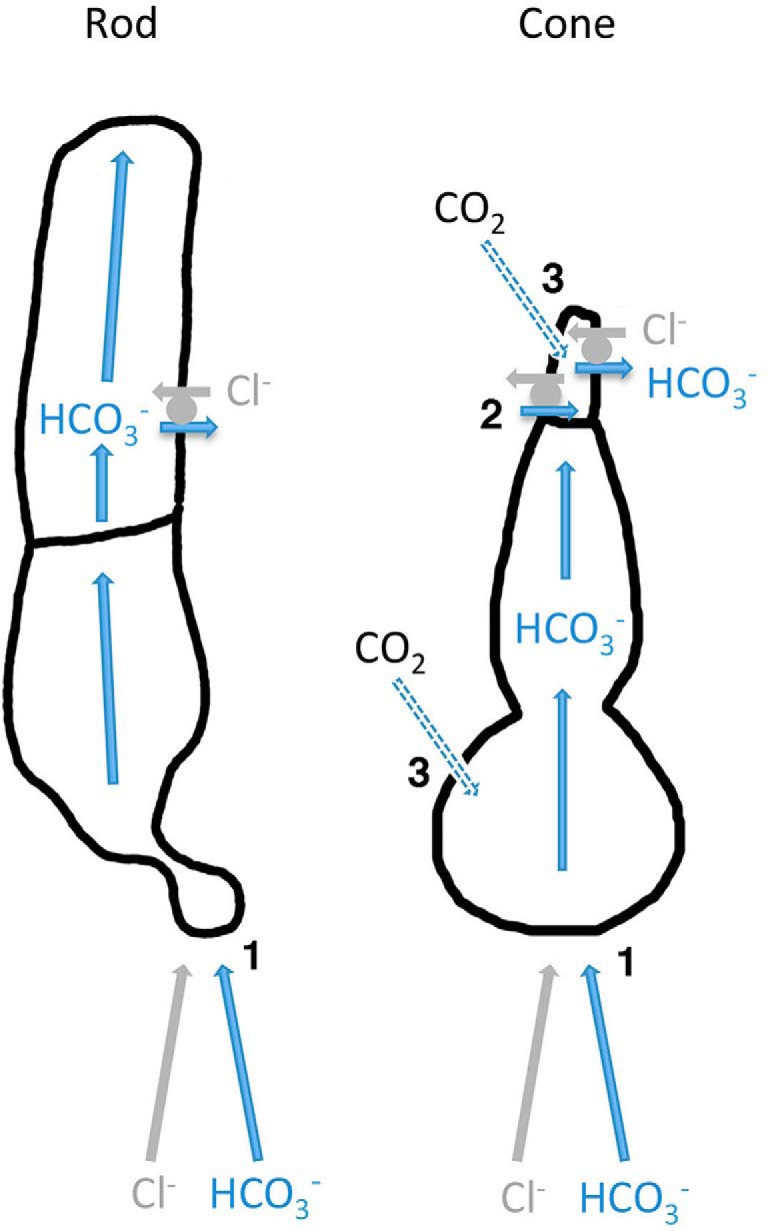
Pathways for bicarbonate trafficking through rods and cones. (1) Bicarbonate enters rods and cones at the synaptic zone through Ca^2+^-activated chloride channels and by Na^+^-dependent transporters. It diffuses through the inner and outer segments and is extruded by an HCO_3_^-^/Cl^-^ exchanger at the OS. (2) In cones, bicarbonate may enter the OS by a reversed action of an HCO_3_^-^ exchanger whose operation seems to be less dependent on external Cl^-^ than the exchanger in rods. (3) Alternatively, bicarbonate can be formed inside the cone by carbonic anhydrase when CO_2_ crosses the membrane. CO_2_ will pass through rods, but the conversion to bicarbonate is slow because rods do not express carbonic anhydrase.

The ability of cone OSs to access bicarbonate in the presence of carbonic anhydrase inhibitor reveals the existence of yet another pathway for entry. Bathing the retina in low [Cl^-^] enhances bicarbonate uptake and produces an even larger circulating current in cones (Koskelainen et al., 1993), presumably by suppressing the exchanger or causing it to load bicarbonate. We lowered [Cl^-^] in the pipette in IS-in recordings to keep [Cl^-^]_i_ low and lowered [bicarbonate] in the bath by half to reduce the driving forces for Cl^-^ leaving the OS and for bicarbonate to enter. Somehow, bicarbonate still found its way into most cones to increase circulating current irrespective of whether carbonic anhydrase inhibitor was present. An HCO_3_^-^/Cl^-^ exchanger is implicated because DIDS blocks the bicarbonate uptake (Koskelainen et al., 1993). It may be that bicarbonate exchange was less dependent on [Cl^-^]_o_ in cones than in rods. We therefore propose at least three routes for bicarbonate entry into cone OSs (Fig. 11). The importance of each pathway may have differed across cells in our experiments in part due to variations in cone morphology and in part because mechanical dissociation led to partial loss of synaptic zones that affected bicarbonate entry, intracellular pH and [Cl^-^]_in_. These multiple pathways may have two functional consequences. First, it may enable cone OSs to accumulate greater levels of bicarbonate than rod OSs. Second, the expression of carbonic anhydrase, which can convert bicarbonate back to CO_2_, and the dependence of the directionality of bicarbonate movement by the exchanger on the bicarbonate gradient could enable cones to stabilize bicarbonate levels within their OSs rather than simply remove it.

Since bicarbonate influences cGMP synthesis by ROS-GC, changes in the production of bicarbonate in retina would be expected to affect visual function. Expression of carbonic anhydrase in red- and green-sensitive cones but not in blue-sensitive cones in humans (Nork et al., 1990) may explain why color vision changes after administration of a carbonic anhydrase inhibitor (Widengård et al., 1995; Leys et al., 1996). Disturbances in bicarbonate levels within the retina arising from defects in metabolism or transport would be expected to give rise to retinal disease due to dysregulation of pH and/or inappropriate ROS-GC activity. Knockout of NBC3 or NBCe2, which might block bicarbonate uptake into photoreceptors, causes a progressive decline in ERG amplitude as rods degenerate (Bok et al., 2003; Kao et al., 2011). The pathologic accumulation of cGMP in retinal rods and cones caused by mutations in phosphodiesterase, ROS-GC, or GCAPs results in degenerative retinal diseases (for review, see Iribarne and Masai, 2017). In such diseases, an overabundance of bicarbonate would be expected to accelerate the course of disease, whereas suppressed bicarbonate levels might have the opposite effect.

## Synthesis

Reviewing Editor: Kirill Martemyanov, The Scripps Research Institute Decisions are customarily a result of the Reviewing Editor and the peer reviewers coming together and discussing their recommendations until a consensus is reached. When revisions are invited, a fact-based synthesis statement explaining their decision and outlining what is needed to prepare a revision will be listed below. The following reviewer(s) agreed to reveal their identity: Peter Calvert, Johan Pahlberg.

Synthesis.

Both reviewers agreed that the study provides extensive body of data on bicarbonate regulation of photoresponses. The reviewers felt that the quality of the results, experimental setup and data analysis were exceedingly excellent. The writing was also deemed clear. We agreed that these results need to be published. However both reviewers felt that more work needs to be done explaining to the readers the significance of the observations, rationale for the study and importantly putting it in a clear context of extensive published literature in this regard. Specifically, it would be necessary to clearly identify what was known before and what new finding the results of the current study add to the picture. Please refer to the specific comments of reviewers for further guidance.

Reviewer's Comments

Reviewer #1

The manuscript eN-NWR-0393-18 explores the mechanism of bicarbonate regulation of the membrane guanylate cyclase in retinal photoreceptors (ROS-GCs). The goal of the work was primarily to identify the sites of bicarbonate entry into photoreceptor cells and to show that bicarbonate directly interacts with ROS-GC to enhance its activity, as opposed to the CO2 associated with bicarbonate. A combination of electrophysiological recordings of rods and cones from tiger salamander retina, using the suction electrode recording technique, and biochemical approaches to measuring ROS-GC activity in cultured COS cells transfected with a vector containing Murine carbonic anhydrase type II and bovine ROS-GC1 cDNAs, or of purified recombinant ROS-GC core catalytic domain, bovine ROS-GC1 G817-Y965. Results showed that ROS-GC stimulation was due to direct interaction between ROS-GC and bicarbonate, and not due to associated CO2. Additionally, physiological experiments where different parts of rods and cones were drawn into the suction electrode, either outer segment in the electrode or inner segment in the electrode, or where parts of isolated cells were sheared off, showed that bicarbonate enters rods only at the synaptic spherule whereas it enters cones at both the spherule and the OS. Moreover, salamander cones were shown to express carbonic anhydrase II throughout their cytoplasm, thus potentially allowing some modulation of bicarbonate levels. The authors are expert physiologists and biochemists and the experimental results are exquisite.

It is hard, however, to suss out where the results differ from what has come before. The results confirm the well-established regulation of ROS-GC by bicarbonate that have been known for decades. They also confirm the previously demonstrated (early 1990s) and well-established Cl- transporter for bicarbonate removal from the cytoplasm. The presence of carbonic anhydrase activity in cones, first demonstrated in the 1970s, was also confirmed in the present study. It is reasonably well established that bicarbonate enters photoreceptors through sodium bicarbonate co-transport by NBC3, found at the synaptic spherule, sodium bicarbonate co-transport through NBCe2 and through Ca2+-activated Cl- channels. The results of the present study do refine the gross localization of these entry routes, which is useful because of difficulties in immunohistochemistry using antibodies to the transporters.

To sum up, the results presented are generally solid and the manuscript is mostly written in a straightforward way. However, it is unclear how the manuscript advances the field. The following are more detailed comments in this regard.

1. The significance statement reads like more of an abstract or summary of findings. It is not clear what the authors think is significant about the work they present in this paper or how it advances the fields of phototransduction or vision.

2. The introduction also could do a better job of setting up how the current work fits into past work on the bicarbonate regulation of ROS-GC and phototransduction. A long paragraph describes the phototransduction cascade in rods in great detail, but an abbreviated paragraph on the roles of bicarbonate barely outlines extensive literature on its influx, efflux, transporters, impact on ROS-GC activity and phototransduction that span decades. Granted, some of this literature is cited later in the paper, but there is no setup in the introduction that would help readers understand how the current work fits into what is already known.

3. The last paragraphs of the discussion section give hints to what is new in the manuscript, that being that cone photoreceptors appear to have three routes for bicarbonate entry. But little is said about why that might be important, except to say that cones may be able to accumulate more bicarbonate in their cytosol, or better stabilize its levels. Why would cones need to do that better than rods? What is the bigger picture?

4. Ultimately, it is not clear from the manuscript why we should be interested in bicarbonate or CO2 levels in the photoreceptor cytoplasm beyond knowing that some amount is needed to maintain normal photoresponse amplitude, kinetics and sensitivity - and whether the authors have substantially added to what is already known.

Reviewer #2

Bicarbonate is abundant in the retina as a metabolic byproduct and, and is know to have effects on photransduction through guanylate cyclase. Since rods and cones are metabolically extremely active, pH buffering becomes important for the physiology of photoreceptors, and the effect of bicarbonate has thus been of interest to investigators for some time.

This manuscript seeks to investigate different modes of bicarbonate entry into photoreceptors, the possible role of CO_2_ in modulating GC activity and photoreceptor physiology, and the difference between rods and cones. Previous studies, eg. by Donner et al 1990, Koskelainen et al 1993, Duda et al 2015, 2018, have shown that bicarbonate acts through GC and differences between the OS and IS of rods and cones exist. Here, the authors conclude that indeed bicarbonate and not CO2 i is affecting phototransduction through GC, carbonic anhydrase is expressed in salamander cones, and cones potentially produce bicarbonate internally through CO2 and carbonic anhydrase. This is a novel, albeit minor, finding which is new in the field.

Some minor concerns exists:

1. The electrophysiological recordings seem to be well executed. However, for a proper flash family more than 3 data points are required, and there is mismatch between graphs showing flash families and intensity response curves. Also, the reported number of recorded cells and the number of cells used for statistics do not match. If the recordings where of poor quality, adding more recordings to increase N would strengthen the statistical analysis.

2. The authors propose 3 ways of accessing bicarbonate in the OS of cones, one of which is production internally through carbonic anhydrase. Did the authors try to separate between the exchanger and CO2 diffusion effect, perhaps pharmacologically, in any other way?

3. Showing more complete flash families for the electrophysiological recordings, especially since they seem to be heavily filtered, would be preferable. Adding to the number of recorded cells for the different conditions would make the conclusions more robust. Separating between the exchanger and diffusion components more robustly would be preferable, although it could be hard to execute. Also, it could be nice to motivate the research question in the abstract and text, and link it a bit stronger to previous literature, to make it more obvious why this is interesting work
